# Measurement of procalcitonin in saliva of pigs: a pilot study

**DOI:** 10.1186/s12917-022-03240-5

**Published:** 2022-04-15

**Authors:** María José López-Martínez, Damián Escribano, Silvia Martínez-Miró, Guillermo Ramis, Edgar G. Manzanilla, Fernando Tecles, Silvia Martínez-Subiela, José J. Cerón

**Affiliations:** 1 Interdisciplinary Laboratory of Clinical Analysis, Interlab-UMU, Regional Campus of International Excellence Campus Mare Nostrum, University of Murcia, 30100 Murcia, Espinardo Spain; 2 Department of Animal Production, Regional Campus of International Excellence Campus Mare Nostrum, University of Murcia, Campus de Espinardo n.17, 30100 Murcia, Espinardo Spain; 3 Pig Development Department, The Irish Food and Agriculture Authority, Teagasc, Moorepark, P61 C996 Fermoy, Co Cork Ireland; 4 School of Veterinary Medicine, University College Dublin, Belfield, Dublin, 4 D04 W6F6 Ireland

**Keywords:** Bacterial infections, Biomarkers, LPS, Meningitis, Porcine, Procalcitonin, Saliva, Sepsis, Turpentine oil

## Abstract

**Background:**

Procalcitonin (PCT) is a widely used biomarker of sepsis in human medicine and can have potential applications in the veterinary field. This study aimed to explore whether PCT could be measured in the saliva of pigs and whether its concentration changes in sepsis. Therefore, a specific assay was developed and analytically validated, and changes in PCT concentration were evaluated in two conditions: a) in an experimental model of sepsis produced by the administration of lipopolysaccharide (LPS) to pigs (*n* = 5), that was compared with a model of non-septic inflammation induced by turpentine oil (*n* = 4), and b) in healthy piglets (*n* = 11) compared to piglets with meningitis (*n* = 20), a disease that usually involves sepsis and whose treatment often requires large amounts of antibiotics in farms.

**Results:**

The assay showed coefficients of variation within the recommended limits and adequate linearity after serial sample dilutions. The method's detection limit was set at 68 μg/L, and the lower limit of quantification was 414 μg/L. In the LPS experiment, higher concentrations of PCT were found after 24 h in the animals injected with LPS (mean = 5790 μg/L) compared to those treated with turpentine oil (mean = 2127 μg/L, *P* = 0.045). Also, animals with meningitis had higher concentrations of PCT (mean = 21515 μg/L) than healthy pigs (mean = 6096 μg/L, P value < 0.0001).

**Conclusions:**

According to these results, this assay could be potentially used as a tool for the non-invasive detection of sepsis in pigs, which is currently a topic of high importance due to antibiotic use restriction.

## Background

Procalcitonin (PCT) is the 13kDa precursor of calcitonin, a hormone with a metabolic role in calcium homeostasis [1, 2]. Almost all PCT is converted into calcitonin in healthy humans, and thus PCT concentrations are in low values in blood [3]. However, in sepsis, PCT is released massively into the bloodstream, and concentrations can rise thousands of fold compared to the physiological values [2, 4–6]. Therefore, its measurement in the blood is widely used in human medicine to diagnose and monitor sepsis and guide antibiotic treatment in bacterial infections, which could be particularly important in the fight against antibiotic resistance [7, 8]. However, there is limited information on the potential applications of this biomarker in veterinary medicine. Particularly in pigs, few studies have been conducted evaluating PCT, and in all cases, this analyte has been measured in serum [5, 9–12].

Saliva is increasingly being used as a diagnostic fluid in animals due to its non-invasive nature and ease of collection by non-specialist personnel. In pigs, saliva is a much more welfare-friendly choice because it avoids the high stress that causes blood collection due to restraining. To date, it has been reported in humans that PCT can be measured in this sample type and may have the potential for detecting sepsis [13]. However, to the authors’ knowledge, PCT has not been measured in the saliva of any other animal species.

This study aimed to investigate if PCT could be analysed in the saliva of pigs and whether its concentration would change in situations of sepsis. For that purpose, the objectives of this study were to develop and validate a specific assay for the measurement of PCT in pig saliva and to evaluate changes in its concentration in two different situations: a) in an experimental model of sepsis by the administration of lipopolysaccharide (LPS) to pigs, which was compared with a model of non-septic inflammation induced by turpentine oil and b) in pigs from a commercial farm with meningitis, which is a condition associated with sepsis and whose treatment is often related to the use of large amounts of antibiotics [14–16]. This article reports the analytical validation of the new assay developed for the measurement of PCT in saliva and the values of PCT in pigs in the different situations above described.

## Results

### Optimisation of the method

**Fig. 1:**
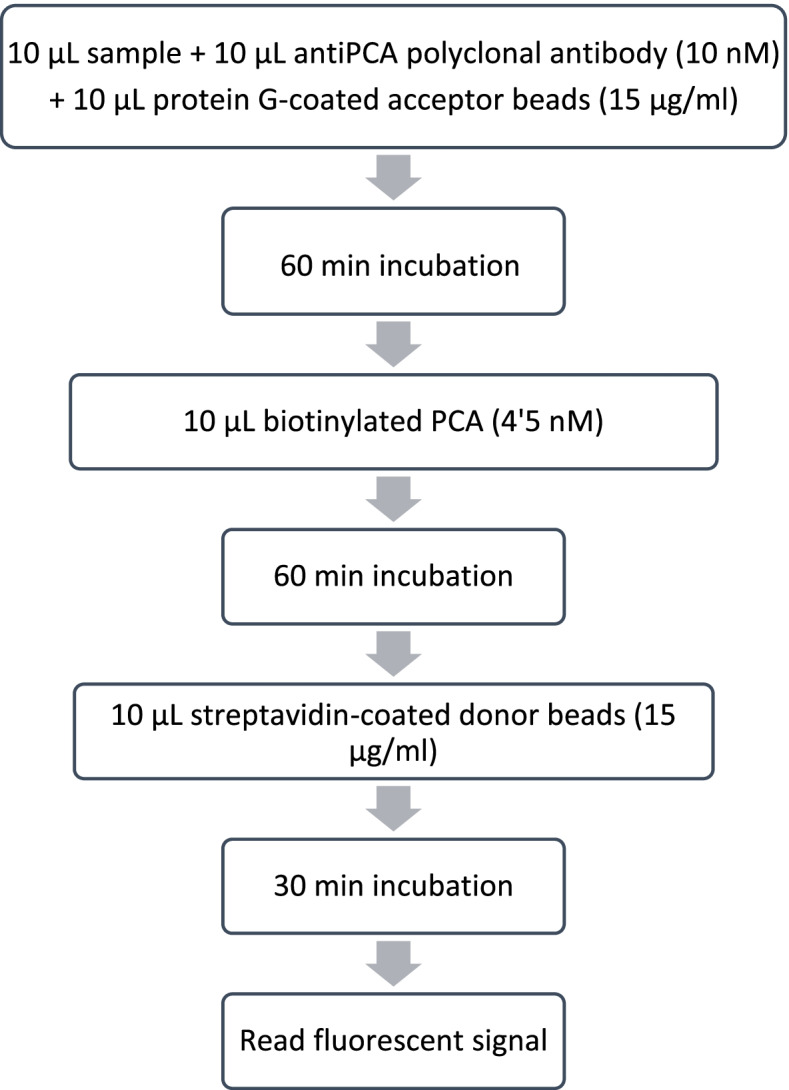
AlphaLISA protocol for PCT measurement in the saliva of pigs

**Fig. 2:**
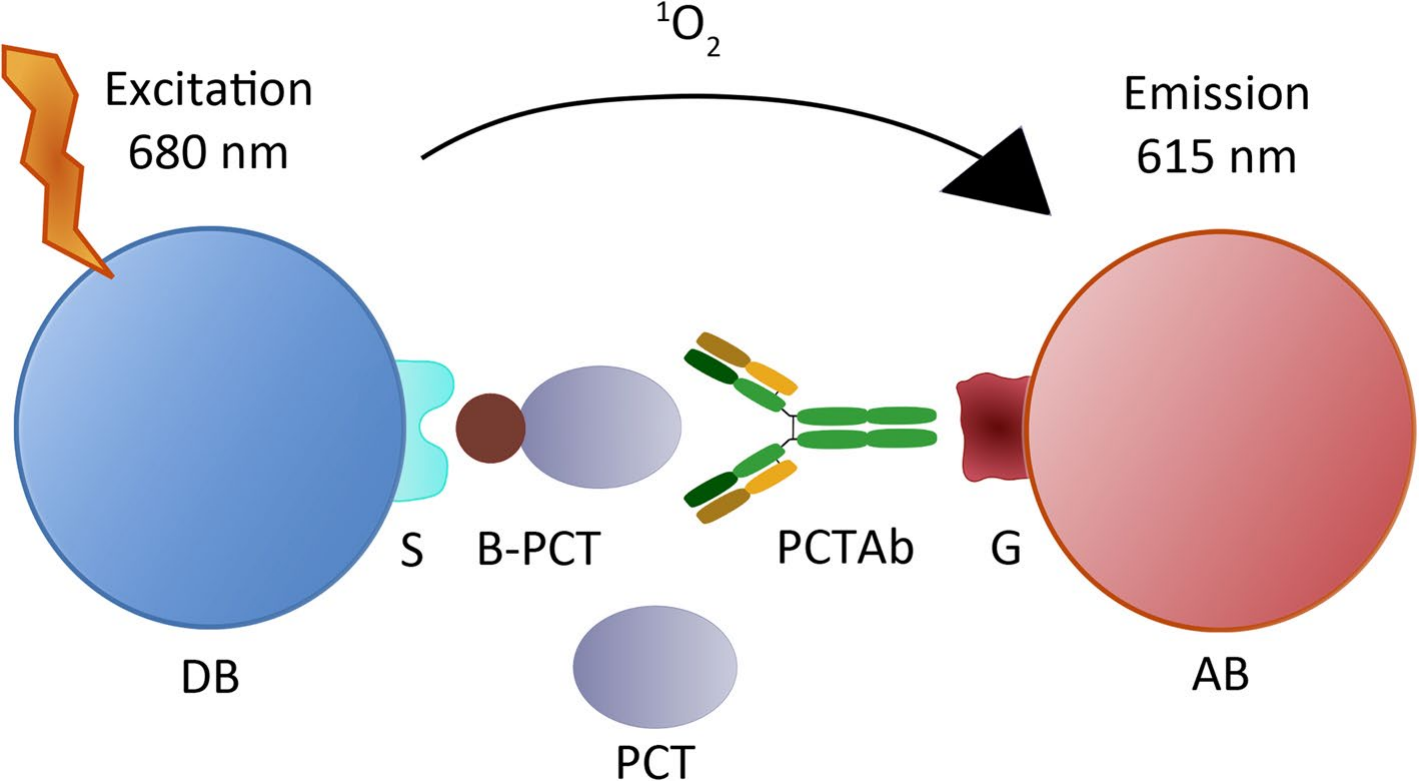
Schematic picture of the AlphaLISA reaction for procalcitonin detection. DB, donor bead; S, streptavidin; B-PCT, biotinylated procalcitonin; PCT, procalcitonin of the sample; PCTAb, Anti-procalcitonin polyclonal antibody; G, protein G; AB, acceptor bead

The optimal concentration of reagents was 4.5 nM for biotinylated PCT, 15 µg/ml for acceptor beads, and 15 µg/ml for donor beads. This combination showed the higher magnitude of signal change before reaching the reaction equilibrium, as well as the maximum buffer signal obtained, and the higher buffer/protein ratio. The final protocol is shown in Fig. 1, and the schematic picture of the AlphaLISA reaction for procalcitonin detection is displayed in Fig. 2.

### Analytical validation of the salivary PCT assay

**Fig. 3:**
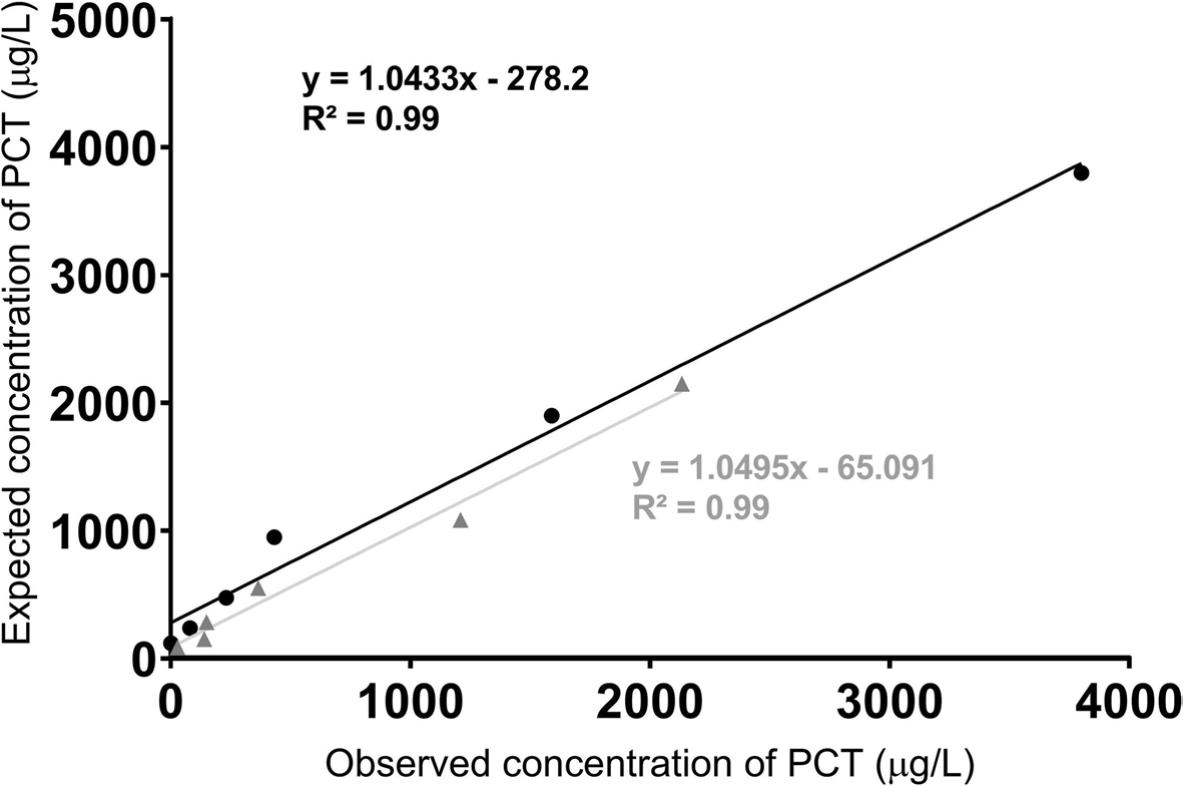
Linearity of dilution of two saliva samples with a high concentration of procalcitonin (µg/L). The obtained salivary procalcitonin concentrations are represented in X-axis, and the expected salivary procalcitonin concentrations are represented in Y-axis. *r*^2^ = coefficient of determination

The intra-assay variation showed CVs of 15.59% and inter-assay CVs of 18.19%. The assay also showed adequate linearity after serial sample dilutions, both with a coefficient of determination of *R*^2^ = 0.99, as represented in Fig. 3. The mean spike recovery test was 88%, between the recommended limits (80–120%) in all cases. The method's LOD was set at 68 μg/L, and the LLOQ at 414 μg/L.

### Changes in salivary PCT after the LPS model

One hour after the injection of LPS, all animals from this group started to show lethargy, increased respiratory rate and depression during approximately 7 h. In addition, one of the animals presented vomiting, and another animal had diarrhoea. The median rectal temperature of the animals after 6 h of the administration of LPS was 41.3ºC.

In the turpentine oil group, the animals showed mild signs of discomfort for several hours but remained alert and active. The median rectal temperature of the animals after 6 h of the administration of turpentine oil was 39.9ºC.

**Fig. 4:**
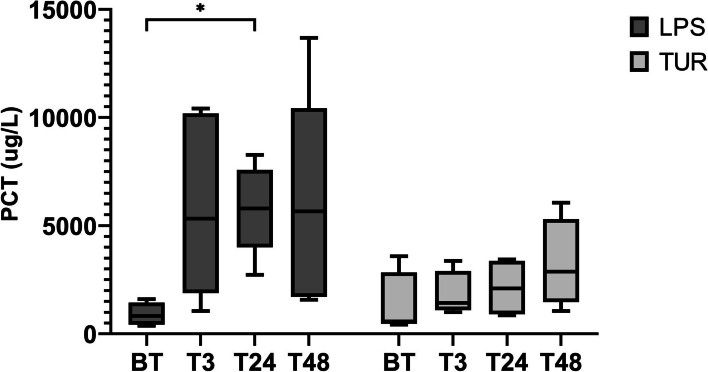
PCT concentrations (µg/L) at the evaluated times in the LPS and turpentine oil. BT = basal time; T3, T24 and T48 = 3, 24 and 48 h after the injections of LPS and oil-turpentine in both groups. Graphs show medians (line within box), 25th and 75th. percentiles (boxes), min and max values (whiskers) and individual values (points). Asterisks indicate statistically significant differences (*P < 0.05)

In the multiple comparisons test, higher concentrations of PCT were found at T24 in the animals injected with LPS (mean ± SD = 5790 ± 2060 μg/L) compared to those treated with turpentine oil (mean ± SD = 2127 ± 1365 μg/L)*, P* = 0.045. The results are graphically represented in Fig. 4.

### Changes in salivary PCT in meningitis

**Fig. 5:**
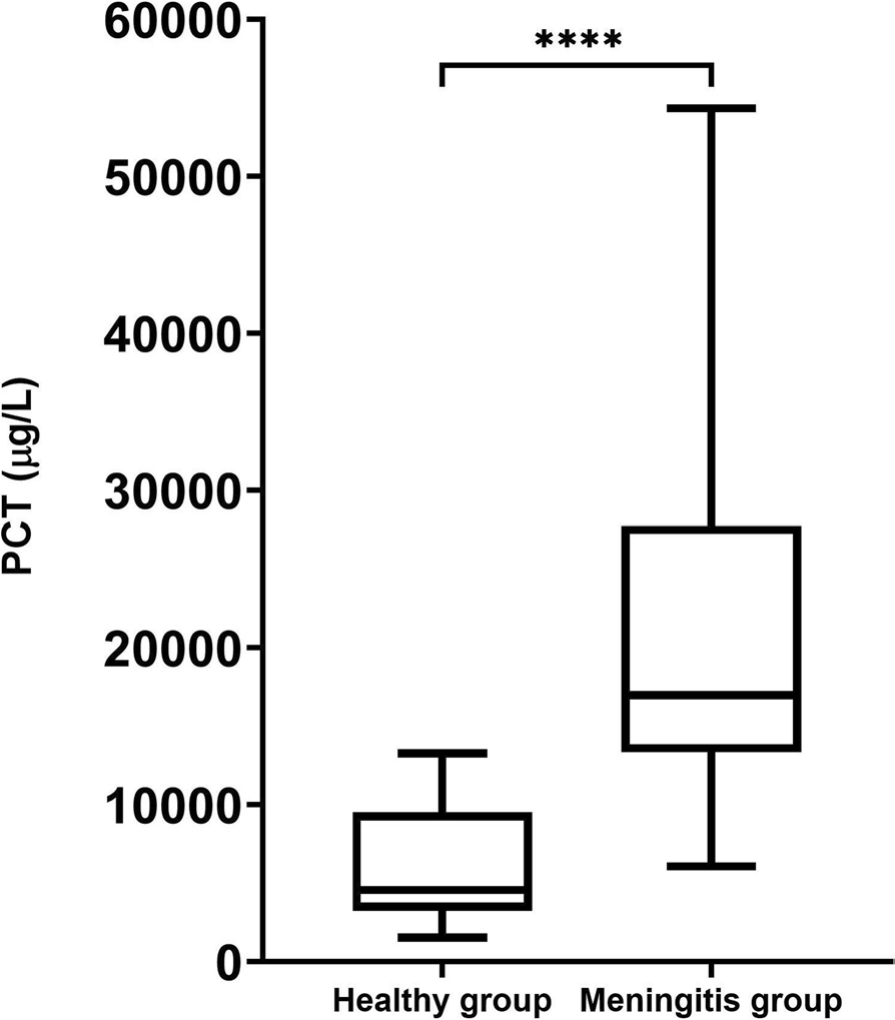
PCT concentrations (µg/L) in the pigs with meningitis compared with the healthy group. Graphs show medians (line within box), 25th and 75th. percentiles (boxes), min and max values (whiskers) and individual values (points). Asterisks indicate statistically significant differences (**** = *P* < 0.0001)

Animals with meningitis had as most frequent symptoms ataxia, anorexia, lateral recumbency, and paddling and a median rectal temperature of 40.5ºC (39.6–40.7ºC, interquartile range). The pigs with meningitis had higher concentrations of PCT (mean = 21515 ± 13289 μg/L) than healthy pigs (mean = 6096 ± 3976 μg/L), with P value < 0.0001. The concentrations of PCT in animals with meningitis vs healthy group are represented in Fig. 5.

## Discussion

This study describes the quantification of PCT in the saliva of pigs for the first time. The assay used was a competitive immunoassay, which has the advantage of detecting antigens regardless of their size, making them helpful for quantifying low molecular weight proteins [17]. This assay showed intra and inter-assay imprecision lower than 20%, which is considered the generally accepted limit [18] and also showed high correlation coefficients and good linearity and spike recovery in serially diluted saliva samples. Therefore, it could be used to measure PCT in porcine saliva. This assay is specific for pigs, and this is important since the homology between human and porcine PCT is low [19], which could explain why human ELISA kits cannot detect in some studies PCT concentrations in pigs [5].

The additional advantage of this assay is the use of AlphaLISA technology, which has shorter incubation times than ELISA and does not need any washing step. In addition, it requires a minimum amount of sample (5 μL) for the analysis, which in the case of pig saliva is especially useful in sick animals, in which it is frequent to obtain small amounts of saliva samples.

The concentrations of PCT in healthy and septic pigs found with this immunoassay were higher than those described in human blood. As this is the first time that PCT has been measured in the saliva of pigs, there are no established reference ranges described in this species and sample. Higher PCT values than humans have been observed in other veterinary species such as horses [20]. Although further studies are needed to understand the reason for these different concentrations, one factor could be the influence in circulating PCT of the species-specific quantities of gram-negative bacteria present in the normal intestinal flora [21, 22]. This factor could explain that in previous reports in horses [23] and our study in pigs, basal concentrations of PCT were detectable, whereas, in humans, basal concentrations of PCT are usually undetectable.

Also, the different magnitude of concentration observed could be due to the type of biological sample or the immunoassay used. For example, in human saliva, two-fold higher concentrations of PCT have been observed compared to serum [13]. Additionally, the antibody used in the assay and its possible affinity to different conformations or states of PCT could lead to differences in immunoassays, as reported with other molecules such as oxytocin [24]. Moreover, PCT is a precursor of the Calcitonin Gene Family of Peptides, which means that other peptides very similar to PCT are eventually released into the bloodstream and could be easily detected by some assays. These peptides have different biological functions, but some share some similarities with PCT, like its possible increase in sepsis, such as adrenomedullin and calcitonin [25, 26].

The experimental model with LPS was used to test the ability of the immunoassay to detect different concentrations of PCT, since higher concentrations of this peptide have been observed in studies with animals treated with LPS [4, 27]. Increases in PCT were also detected in the group treated with turpentine oil, which produces a non-septic inflammation. However, these increases were of lower magnitude compared with the LPS group. For example, the pigs with the LPS administration had a mean 6.35-fold increase at T24 compared to TB, whereas at T24, the pigs with the turpentine oil administration had a mean 1.64-fold increase compared to TB. These increases in PCT in non-septic inflammatory conditions have been described in humans in conditions like severe burns, trauma, or major surgery. These increases are usually of less magnitude [28], similarly to what occurs in our study, and therefore, in human medicine, the use of cut-off points that help differentiate diseases caused by sepsis and non-septic inflammatory conditions is standardised [29].

At the farm level, the piglets with meningitis in our study had a mean 3.53-fold increase in PCT concentrations compared to healthy piglets. The magnitude of increases in PCT obtained in our study in sepsis is consistent with previous studies performed in human saliva. For example, a 3.45-fold increase in salivary PCT was observed in exacerbations of bacterial origin in Chronic Obstructive Pulmonary Disease [13]. In our study, the magnitude of increase of PCT in piglets with meningitis compared to healthy ones was lower than in the LPS group. These variations could be related to the differences in severity and duration over time of the two conditions. They could also be influenced by differences based on age, as in human medicine, PCT average concentrations are higher in neonates [30], and in our study, the piglets from the healthy/meningitis group were younger than the pigs from the LPS model. Therefore, reference ranges for different pathologies and ages should be established in the future in the case of PCT in saliva. Also, PCT levels in saliva could be influenced by the health status of the farm, a factor that should be studied in more detail in future research.

The main limitation of this report is the number of animals used; consequently, this should be considered a pilot study. In addition, although the target of the study was to validate the method in a non-invasive sample that did not generate stress on the animals, another limitation would be the lack of comparison with other biological samples such as serum. A correlation was reported between procalcitonin in saliva and serum from humans [31], but there is no data in the pig. Therefore, further research would be of interest to validate this assay in other potential samples. In addition, a more significant sample and a more extensive range of pathologies at the farm level are necessary to set optimal cut-offs points, ideally at different ages, to differentiate between animals with and without sepsis. Another limitation of this study is that it cannot be ensured that in our work conditions, the streptococcal meningitis was not accompanied by other concomitant pathologies that can occur in this disease [32].

## Conclusions

A precise and accurate assay has been developed to quantify PCT in the saliva of pigs. This assay was applied to the saliva of pigs with experimentally induced sepsis and non-septic inflammation, and the increases were higher in the septic model. The assay also detected higher values of PCT in the saliva of piglets with meningitis compared to healthy piglets. Therefore, this assay could be potentially used as a tool for the non-invasive detection of sepsis in pigs, which is currently a topic of high importance due to antibiotic use restriction.

## Methods

### Development and optimisation of the assay for PCT measurement

#### Antibody production

Antibodies were produced according to standard protocols (University of California Berkley Animal Care and Use Committee, 2009) in a New Zealand rabbit (female, 2.5 kg, 3-months old) supplied by the commercial farm Granja San Bernardo (Navarra, Spain). The rabbit was immunised using 100 μg of porcine PCT (Biovendor, RD572451100) as an antigen, diluted in NaCl and emulsified in Freund’s adjuvant (complete in the first immunisation, incomplete in the booster ones) in a total of 0.2 ml subcutaneously [33]*.* A week after each immunisation, blood was collected via the auricular vein of the rabbit, and serum was screened through ELISA to evaluate the antibody titration. After the final blood collection, the rabbit was anaesthetised with intramuscular xylazine at a dose of 3–9 mg/kg. Then, when the rabbit reached unconsciousness, it was euthanised by barbiturate overdose through intravenous administration of sodium pentobarbital at a dose of 150 mg/kg.

#### Antibody purification

To avoid interferences of the antibodies with other compounds, they were purified with an automated liquid chromatography system (ÄKTA pure, GE Healthcare Life Sciences), passing the rabbit serum through a HiTrap protein G HP affinity column according to the manufacturer’s instructions (GE Healthcare Life Sciences, Munich, Germany).

#### PCT biotinylation

PCT was biotinylated with the commercial kit EZ-Link™, Micro Sulfo-NHS-Biotin, No-Weight™ Format (Thermo Scientific, USA) with a 50-fold molar excess following the manufacturer’s instructions.

#### Development and optimisation of AlphaLISA method

AlphaLISA technology (PerkinElmer, Inc., MA, USA) allows the development of amplified luminescent proximity homogeneous assays that provide several advantages over other similar assays, such as the no need to wash the plate or the use of minimal sample quantities. An indirect competitive assay was developed for PCT measurement, which can be performed in 96-well plates (PerkinElmer, Inc., MA, USA) with a total volume of 50 uL per well. In order to optimise assay conditions, different concentrations of all components were evaluated. The performance of each combination was tested with a constant amount of procalcitonin (1000 ng/ml) and assay buffer used as a blank. Then, the magnitude of signal change (expressed as counts in AlphaLISA assays), the maximum signal obtained and the buffer/protein ratio were evaluated with each condition. The combinations that were tested included 0, 0.3, 3, 4.5 and 6 nM of biotinylated PCT; 10 and 15 nM of polyclonal antiPCT antibody; 5, 10, 15 and 20 µg/ml of Donor beads coupled to streptavidin; and 5, 10, 15 and 20 µg/ml of Acceptor beads coupled to protein G (PerkinElmer, Inc., MA, USA). In addition, several samples with high and low concentrations of procalcitonin were diluted ranging from 1:2 to 1:16 to assess which dilution showed best linearity. As a standard, a commercial porcine PCT (Biovendor R&D, Brno, Czech Republic) was used, and the curve was conducted with concentrations ranging from 10 to 10000 ng. Finally, the performance of three different buffers (PBS, alpha buffer and universal buffer, the last ones from PerkinElmer, Inc., MA, USA) was tested with the standard and several samples. Results were expressed in μg/L.

### Analytical validation of the AlphaLISA method

#### Imprecision

Imprecision was assessed through intra and inter-assay coefficients of variation (CVs), calculated as the standard deviation divided by the mean of the values of the different replicates multiplied by 100. The intra-assay imprecision was calculated by measuring five replicates of samples with a high, medium, and low concentration of PCT at the same time. The assessment of the inter-assay imprecision was performed by the measurement of five aliquots of each saliva sample that were stored at -80ºC, and each one was analysed in duplicate along five different days. All the samples used in the analytical validation were obtained from the LPS experimental model described in this article.

#### Accuracy

Accuracy was tested by assessing the linearity of serial sample dilution. Also, the matrix effect was tested through spike and recovery tests as previously reported [34, 35]. Two samples with a high PCT concentration were serially diluted from 1:4 to 1:128 with the assay buffer for the linearity assessment. The recovery experiment was performed by adding different concentrations of porcine PCT standard (10000, 5000, 3000, 1000, 300 and 10 ng) to a diluted (1:4) porcine saliva sample with a low concentration of PCT.

#### Sensitivity

Sensitivity was assessed by the limit of detection (LOD) and the lower limit of quantification (LLOQ). LOD was calculated as the mean value of 12 replicate PCT determinations of the assay buffer plus three standard deviations (SD). In contrast, LLOQ was evaluated by serially diluting a porcine saliva sample with the assay buffer and analysing five replicates of each dilution. Then, CVs of each dilution were calculated, and LLOQ was set on the lowest PCT concentration that could be repeatedly measured with a 20% CV or lower.

### Changes in PCT in an LPS experimental model

#### Animals

Growing male pigs [(*Sus scrofa domesticus) (Large White)]* in the mid-fattening period from the Experimental Farm of the University of Murcia (Murcia, Spain) were used to perform the experimental model. On this farm, pigs are kept from birth until they are sent to the slaughterhouse at about 24 weeks old. At the moment of sampling, pigs were 14 weeks old and had a median weight of 51.5 kg (interquartile range 48–53 kg). Pigs were given *ad libitum* access to a nutritionally balanced diet and water and were housed with a minimum space of 0.65 m^2^ per animal (Council Directive 2001/88/CE of 23 October 2001 amending Directive 91/630/CEE concerning minimum standards for the protection of pigs) and an average temperature of 24 ± 2 °C. 

#### Groups and sample collection

A total of nine animals were chosen at random by convenience sample and were divided into two separate groups for the experimental model. Animals were adapted to experimental conditions (groups, housing, diet, and ambient temperature) for one week before starting the experiment. All animals and samples obtained were appropriately identified to avoid potential confounders. No prior potential animal exclusion criteria were established, and there were no later exclusions from the study. All participants were aware of the location of the two groups during all phases of the experiment. The first group (*n* = 5) was administered LPS from *Escherichia Coli (LPS; O55:B5, Sigma-Aldrich)* reconstituted in sterile saline solution in a single dose of 30 ug/kg per animal by intramuscular route [36]. In the second group (*n* = 4), the animals were treated with a total of 8 mL subcutaneous injections of turpentine oil (oil of turpentine purified, Sigma–Aldrich), 4 mL in each front flank per animal, as previously described [37].

The administration of the compounds was performed between 8–9 am, and based on the time of the injection of each animal, 4 sample collection times were established: BT (basal time, 24 h before the LPS and turpentine oil injections), used as a control sample; and T3, T24 and T48 (3, 24 and 48 h after the respective intramuscular injections). At each time, saliva samples from all the animals were collected. Additionally, rectal temperature was measured 6 h after administration of both compounds.

Saliva was collected using a sponge clipped to a flexible thin metal rod approximately 20 cm in length. Pigs were allowed to chew on the sponge until thoroughly moist, and then, the sponges were introduced in *Salivette* tubes (Sarstedt, Aktiengesellschaft & Co. D-51588 Nümbrecht, Germany). All samples were kept refrigerated until arrival at the laboratory, where the *Salivette* tubes were centrifuged at 3000* g* and 4ºC for 10 min to obtain saliva. Then, samples were transferred into Eppendorf tubes and stored at -80ºC until analysis.

### Changes of PCT in meningitis

#### Animals

Weaning pigs [(*Sus scrofa domesticus) (Large White)*] from 6 to 9 weeks old from a commercial farm located in the same geographical area as the University of Murcia (Region of Murcia, south-eastern Spain) were chosen at random by convenience sample*.* Two groups were established, integrated by clinically healthy pigs (*n* = 11) belonging to one pen and pigs diagnosed with meningitis (*n* = 20) belonging to another pen. All animals and samples obtained were appropriately identified to avoid potential confounders. The animals with meningitis had clinical symptomatology compatible with this disease [32] and had been recently positive to *Streptococcus suis* in bacteriological cultures performed in blood agar plates following standard procedure [38]. After the study, the animals were kept in the farm under the standard production management. No prior potential animal exclusion criteria were established. There were no later exclusions from the study. All participants were aware of the location of the two groups during all phases of the experiment.

#### Sample collection

Saliva was collected, processed and analysed as described in the previous subsection Groups and sample collection.

### Statistical analysis

Descriptive statistics and linear regression equations were calculated using routine descriptive statistical procedures and computer software (Excel 2016, Microsoft). Statistical analysis and graphs were performed using RStudio software (RStudio Team (2019). RStudio: Integrated Development for R. RStudio, Inc., Boston, MA URL http://www.rstudio.com/) and Graph Pad software (GraphPad Prism, version 9 for Windows, Graph Pad Software Inc., San Diego, USA).

The LPS and turpentine oil experiment data were evaluated for normality of distribution using the Shapiro–Wilk test and showed a non-normal distribution; therefore, data were log-transformed and then analysed with a linear mixed model followed by a multiple comparisons test*.* The data from the healthy piglets and piglets with meningitis were evaluated for normality of distribution using the Shapiro–Wilk test and showed a non-normal distribution; therefore, data were log-transformed and evaluated with an unpaired-two sample t-test to evaluate differences between both groups. Results were reported as mean ± SD of results expressed as µg/L of PCT and represented in box and whiskers plots in Figures. The alpha level for determination of significance was 0.05.

## Data Availability

The datasets used and/or analysed during the current study are available from the corresponding author on reasonable request.

## Abbreviations

PCT: Procalcitonin
LPS: Lipopolysaccharide
CVs: Coefficients of variation
LOD: Limit of detection
LLOQ: Lower limit of quantification
SD: Standard deviation
